# The Diabetic Nose: A Narrative Review of Rhinologic Involvement in Diabetes (1973–2025)

**DOI:** 10.3390/jcm15020472

**Published:** 2026-01-07

**Authors:** Giulio Cesare Passali, Mariaconsiglia Santantonio, Desiderio Passali, Francesco Maria Passali

**Affiliations:** 1 Complex Operational Unit of Ear, Nose and Throat Sciences, Fondazione Policlinico Universitario A. Gemelli, IRCCS–Scientific Institute for Research, Hospitalization and Healthcare, 00168 Rome, Italy; giuliocesare.passali@unicatt.it; 2 Department of Mental Health and Sensory Organs, University of Siena, 53100 Siena, Italy; d.passali@virgilio.it; 3 Department of Clinical Sciences and Translational Medicine, Otorhinolaryngology Unit, Tor Vergata University of Rome, Via Cracovia 50, 00133 Rome, Italy; passali@med.uniroma2.it

**Keywords:** diabetes mellitus, chronic rhinosinusitis, olfactory dysfunction, sinusitis, rhinitis, rhinology, DM

## Abstract

**Background**: Although diabetes mellitus is traditionally viewed as a systemic metabolic disorder, growing evidence indicates that it also affects the upper airways through vascular, inflammatory, and neuro-sensory mechanisms. The sinonasal mucosa, highly vascularized and immunologically active, may represent an early target of diabetic microangiopathy and immune–metabolic imbalance. **Objectives**: Our objectives are to synthesize current evidence on the rhinologic manifestations of DM, with a focus on chronic rhinosinusitis, olfactory dysfunction, and other nasal disorders, and to identify the main pathophysiologic and clinical patterns linking diabetes to sinonasal disease. **Results**: Evidence suggests that DM, particularly type 2 DM, increases susceptibility to CRSwNP and modulates the sinonasal microbiome toward Gram-negative predominance. Surgical outcomes after endoscopic sinus surgery are generally comparable between diabetics and non-diabetics when perioperative care is optimized. Olfactory dysfunction occurs more frequently and severely in diabetic patients, likely reflecting the combined effects of chronic inflammation, vascular compromise, and insulin resistance. Additional manifestations include recurrent epistaxis, delayed mucociliary clearance, and chronic cough. Allergic rhinitis appears to not be increased, and maybe even inversely related, especially among users of DPP-4 inhibitors. **Conclusions**: Diabetes intersects with rhinologic health through immune–metabolic, vascular, and epithelial pathways that may shape susceptibility, disease phenotype, and neurosensory decline. Future research should focus on disentangling type-specific mechanisms, metabolic biomarkers, and longitudinal outcomes, with the aim of developing precision-based approaches to rhinologic assessment and management in diabetic patients.

## 1. Introduction

### 1.1. Diabetes Mellitus Type 1

Type 1 diabetes mellitus (T1D or T1DM) is an autoimmune disease characterized by the progressive destruction of pancreatic β-cells by autoreactive CD4^+^ and CD8^+^ T lymphocytes and macrophages, leading to absolute insulin deficiency [1,2,3,4,5]. It accounts for approximately 5–10% of all diabetes cases worldwide [2] and remains the predominant form in childhood and adolescence, although a considerable proportion of diagnoses occur in adulthood. The 2024 global prevalence was estimated at around 9 million individuals, including 1.8 million under the age of 20 [1].

The incidence of T1DM shows remarkable geographic variability, with extremely high rates in Northern European countries and very low rates in Asia and South America (up to 350–400-fold differences between regions) [4,5]. According to the WHO DIAMOND Project and the EURODIAB study, the incidence among children under 15 years ranges from 0.1 per 100,000 in China and Venezuela to over 36 per 100,000 in Finland.

Over recent decades, the incidence of T1DM has been rising globally by approximately 2–5% per year, with the steepest increases observed among young children and in regions previously classified as low-incidence. This rapid rise cannot be explained by genetic changes alone and likely reflects environmental influences acting upon susceptible genotypes.

From a pathogenetic standpoint, T1DM progresses through distinct stages: two asymptomatic preclinical phases (stages 1 and 2) characterized by the appearance of multiple islet autoantibodies against β-cell antigens such as GAD65, IA-2, and insulin, followed by stage 3, marked by hyperglycaemia and insulin dependence [1].

Overall, T1DM is a chronic, immune-mediated condition with a steadily increasing incidence worldwide and substantial implications for public health due to its acute and chronic complications [6,7,8,9]. Its multifactorial aetiology, at the intersection of genetics, immune dysregulation, and environmental exposure, continues to be a major focus of translational and epidemiological research.

### 1.2. Diabetes Mellitus Type 2

Type 2 diabetes mellitus (T2D or T2DM) is a complex, multifactorial metabolic disorder characterized by chronic hyperglycaemia resulting from a combination of insulin resistance and a progressive decline in pancreatic β-cell function [10,11,12,13]. It represents approximately 90% of all diabetes cases globally and is increasingly diagnosed across all age groups, including adolescents [13]. According to the International Diabetes Federation, in 2021 there were an estimated 537 million adults living with diabetes worldwide, with projections suggesting a rise to 783 million by 2045 [14]. T2DM is therefore considered one of the “big four” non-communicable diseases alongside cardiovascular disease, cancer, and chronic respiratory disease.

The aetiology of T2DM involves a complex interplay between genetic, environmental, and lifestyle factors. Obesity remains the most significant modifiable risk factor, contributing to more than 50% of global disability-adjusted life years (DALYs) related to T2DM. Excess caloric intake, high-fat and high-sugar diets, and sedentary behaviour promote adipose tissue dysfunction, lipotoxicity, and low-grade inflammation, which collectively drive insulin resistance and β-cell failure [15,16]. At the cellular level, mitochondrial dysfunction, oxidative stress, and endoplasmic reticulum stress further exacerbate insulin resistance and impair glucose metabolism.

Genetic predisposition contributes substantially to T2DM risk, with heritability estimates ranging from 30 to 70% [17,18].

Epidemiological studies show that T2DM disproportionately affects low- and middle-income countries, where more than 80% of cases occur [19]. The prevalence rises markedly with age and is slightly higher in men, though women face a greater lifetime risk. Sex hormones and body-fat distribution contribute to these differences: men typically develop T2DM at a lower BMI but with greater visceral fat, whereas women show stronger associations between obesity and diabetes risk. In addition to obesity and genetics, psychosocial and behavioural factors such as socioeconomic disadvantage, chronic stress, poor diet, and sleep deprivation play important roles in disease onset and progression.

From a mechanistic perspective, T2DM is not a uniform disease entity but a heterogeneous spectrum encompassing variable degrees of β-cell dysfunction and insulin resistance [20,21].

### 1.3. An Overview of Nasal Disorders

#### 1.3.1. Inflammatory and Infectious Disorders

Allergic rhinitis (AR) and chronic rhinosinusitis (CRS) represent the most common chronic inflammatory diseases of the upper airways, while acute and fungal rhinosinusitis illustrate their infectious spectrum.

AR is an IgE-mediated Th2-driven disorder characterized by eosinophilic inflammation, epithelial barrier dysfunction, and cytokine release (IL-4, IL-5, IL-13). Beyond Th2, lymphocytes, ILC2, Tfh, and regulatory T/B cells contribute to chronicity through impaired IL-10–mediated tolerance. Allergen-specific immunotherapy remains the only disease-modifying treatment, and biologics targeting IgE or IL-4Rα benefit severe cases [22,23,24,25,26].

CRS, defined by sinonasal inflammation persisting > 12 weeks, is divided into CRS with nasal polyps (CRSwNP) (type 2/eosinophilic) and CRS without nasal polyps (CRSsNP) (type 1/3/neutrophilic) forms. Barrier dysfunction, reduced mucociliary clearance, and microbial dysbiosis sustain chronicity, while *Staphylococcus aureus* colonization and persistent tissue-resident Th2/Th17 cells drive recurrence despite corticosteroid therapy or surgery [27,28,29,30].

Acute bacterial sinusitis (ABS) is a secondary bacterial complication of viral infections, affecting ~2% of adults and up to 13% of children. It is linked to mucosal edema and impaired mucociliary clearance that facilitate growth of Streptococcus pneumoniae and *Haemophilus influenzae*. Diagnosis is mainly clinical, and antibiotic therapy is reserved for confirmed bacterial cases [31,32,33,34,35,36].

Fungal rhinosinusitis (FRS) includes non-invasive forms—saprophytic infestation, fungal ball, and allergic fungal rhinosinusitis (AFRS)—and invasive forms (acute, chronic, granulomatous). AFRS occurs in atopic, immunocompetent hosts as a Th2-mediated allergic response to dematiaceous fungi, while invasive forms affect immunocompromised or diabetic patients, causing angioinvasion, tissue necrosis, and high mortality, predominantly due to *Mucorales* or *Aspergillus* species [37,38,39].

Overall, these conditions exemplify how immune dysregulation, epithelial dysfunction, and microbial imbalance interact to sustain sinonasal inflammation, pathways that may be further amplified in diabetes through vascular injury, oxidative stress, and impaired host defence.

#### 1.3.2. Olfactory Dysfunction

Olfactory dysfunction (OD) affects up to 20% of the population and arises from diverse causes including inflammatory sinonasal disease, viral infections, head trauma, neurodegenerative disorders, and metabolic conditions such as diabetes. The olfactory mucosa functions as both a sensory and immune barrier, containing specialized epithelial, neuronal, and immune cells. The not very frequently mentioned blood–olfactory barrier (BOB) restricts systemic antibody access, rendering the olfactory mucosa particularly vulnerable to pathogens and inflammation [40,41,42,43,44].

Local immune responses involving macrophages, T cells, and plasma cells protect against infection but may also contribute to chronic smell loss. OD thus reflects a complex interaction between immune regulation, epithelial integrity, and neural function, mechanisms that may be further impaired in metabolic diseases such as diabetes.

Despite the high global burden of DM, knowledge of its rhinologic manifestations remains limited and methodologically inconsistent. Existing studies differ substantially in diabetes classification, rarely distinguish between type 1 and type 2 disease, and apply non-uniform criteria for sinonasal outcomes, particularly for OD, which is often assessed subjectively rather than through validated testing.

Consequently, the overall clinical significance of diabetes-related rhinologic involvement remains incompletely defined.

Against this background, the present narrative review aims to critically synthesize the available human adult literature published between 1973 and 2025 and indexed in PubMed on the association between diabetes mellitus and rhinologic disorders.

## 2. Materials and Methods

### 2.1. Research and Screening of the Literature

A comprehensive literature search was conducted to explore the association between diabetes mellitus (type 1 and type 2) and rhinologic diseases with a predominant vascular or immunological component. The search was performed in PubMed, covering the period from 1973 to 2025. The following Boolean string was used:

*(“diabetes mellitus” OR “diabetes” OR “type 1 diabetes” OR “type 2 diabetes” OR “DM1” OR “DM2” OR “insulin-dependent diabetes” OR “non-insulin-dependent diabetes”)*

*AND*

*(“rhinitis” OR “allergic rhinitis” OR “non-allergic rhinitis” OR “vasomotor rhinitis” OR “atrophic rhinitis” OR “chronic rhinitis” OR “acute rhinitis” OR “infectious rhinitis” OR “sinusitis” OR “chronic sinusitis” OR “rhinorrhea” OR “nasal obstruction” OR “nasal congestion” OR “nasal discharge” OR “nasal dryness” OR “nasal crusting” OR “nasal polyps” OR “nasal inflammation” OR “nasal infection” OR “nasal mucosa” OR “nasal complications” OR “nose disease” OR “nose diseases” OR “sinonasal disease” OR “sinonasal inflammation” OR “olfactory dysfunction” OR “olfactory disorder” OR “olfactory impairment” OR “olfactory loss” OR “smell disorder” OR “smell loss” OR “anosmia” OR “hyposmia” OR “dysosmia” OR “parosmia” OR “phantosmia”)*

PubMed was chosen as the primary database because it represents the most authoritative and widely used source for biomedical and clinical research, offering extensive coverage of the peer-reviewed literature in endocrinology and otorhinolaryngology. The use of *MeSH* (Medical Subject Headings) terms allows precise identification of clinically relevant studies, which was aligned with the narrative- and pathophysiology-oriented scope of this review.

Without applying any filters, the Boolean search string retrieved 961 records from PubMed, including preprints, non-English publications, animal studies, and studies involving pediatric populations.

Database filters were then applied to exclude duplicate records and review articles and to restrict the search to human studies. Although filters for adult populations were applied, animal and pediatric studies were not always reliably excluded due to database indexing limitations.

These records were therefore excluded during a first manual screening based on titles and abstracts, together with studies unrelated to rhinologic or diabetic outcomes.

**Figure 1:**
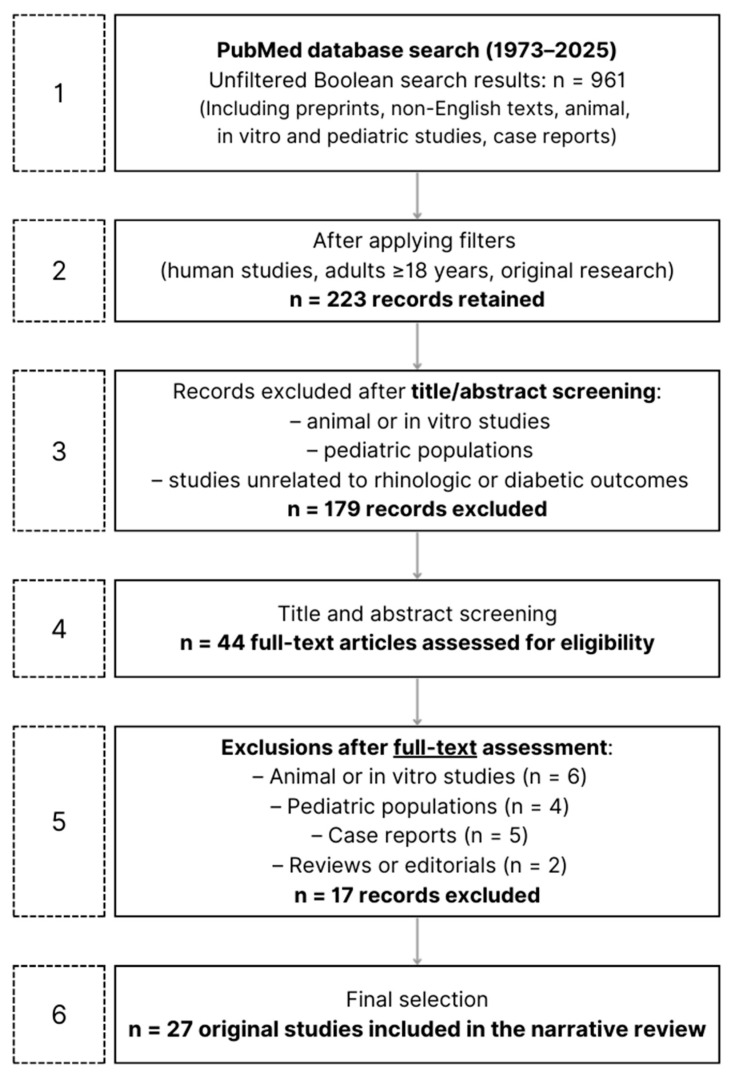
Flow diagram summarizing the literature search strategy and study selection process. The initial unfiltered Boolean search on PubMed (1973–2025) yielded 961 records, including preprints, non-English texts, animal, in vitro, and pediatric studies, and case reports (Step 1). After application of database filters to restrict the search to human studies, adults ≥ 18 years, and original research, 223 records were retained (Step 2). Records were then screened by title and abstract, and studies clearly unrelated to rhinologic or diabetic outcomes, as well as animal/in vitro and pediatric studies identifiable at this stage, were excluded (Step 3), resulting in 44 full-text articles assessed for eligibility (Step 4). A subsequent manual full-text assessment led to the exclusion of additional studies, including animal or in vitro studies, pediatric populations, case reports, and reviews or editorials, when the study design or population was not clearly identifiable from the title or abstract alone (Step 5). Following this process, 27 original studies were included in the final narrative review (Step 6).

This screening yielded 44 articles eligible for full-text assessment. Among these 44 articles, 17 studies were excluded only after full-text assessment because the study design or population (animal, in vitro, or pediatric) was not clearly identifiable from the title or abstract, which primarily reported outcomes in adult human populations (Figure 1).

Since the literature exploring the vascular and immunological interplay between diabetes and rhinologic disorders remains limited and heterogeneous, an extended time frame (1973–2025) was selected to encompass both early pathophysiological reports and contemporary studies, thereby providing a comprehensive overview of the topic’s evolution over time. Given the heterogeneity of study designs and outcome measures, a narrative synthesis approach was adopted, focusing on mechanistic and clinical evidence linking diabetes-related metabolic, vascular, and immune alterations to rhinologic manifestations. Selected studies were qualitatively analyzed and grouped into predefined thematic categories according to the main rhinologic domain investigated. Within each category, findings were descriptively compared with attention to study design, population characteristics, diabetes phenotype (when specified), and proposed immunological, vascular, or metabolic mechanisms. No quantitative synthesis or formal assessment of risk of bias was performed.

### 2.2. Inclusion and Exclusion Criteria

Inclusion criteria comprised original research (clinical, observational, or experimental) investigating any form of DM (type 1 or type 2) in association with rhinologic or sinonasal diseases in adult human populations (≥18 years). Studies were required to provide vascular, immunological, or inflammatory data relevant to nasal or sinonasal pathology. Exclusion criteria included animal or in vitro studies, paediatric cohorts, review articles, editorials, case reports, and studies unrelated to the investigated association.

## 3. Review of the Literature

To facilitate comprehension, the literature review was organized into three main sections: inflammatory and infectious rhinologic disorders, olfactory dysfunction, and other miscellaneous manifestations involving the nasal and sinonasal tract.

### 3.1. Inflammatory and Infectious Rhinologic Disorders

Across inflammatory conditions, DM shows a selective and often nuanced relationship with sinonasal disease. In a large, population-based analysis published in 2022, the authors of [45] identified a robust association between DM and CRSwNP—but not with CRSsNP—and noted more prolonged smell dysfunction among diabetic patients with CRS, aligning with the broader concept that immune–metabolic dysregulation in diabetes can amplify chronic inflammation and susceptibility to infection. Extending this inflammatory–infectious axis into the operative setting, a study from 2014 [46] reported that CRS patients with diabetes harbour a distinct sinus microbiologic signature, enriched for *Pseudomonas aeruginosa* and other Gram-negatives, and experience less quality-of-life improvement six months after FESS, despite similar preoperative severity. Together, these findings suggest that diabetic terrain may shape both the inflammatory phenotype (polyposis) and the infectious ecology (Gram-negative predominance) of CRS.

Surgical outcomes themselves, however, are not uniformly worse in diabetes. In a matched nested case–control study of medically recalcitrant CRS from 2015 [47], comparable postoperative gains in symptoms and quality of life between diabetic and non-diabetic patients were found, with neither insulin use nor glycaemic indices predicting outcome. These data imply that when perioperative care is optimized, diabetes per se does not preclude meaningful benefit from ESS, even if microbiological patterns differ.

A prospective multicentre cohort study [48] of 241 CRS patients undergoing ES examined the impact of obesity and diabetes on surgical outcomes. Diabetes was more frequent in overweight and obese individuals but did not preclude significant postoperative quality-of-life improvement. However, obese patients showed a smaller relative gain. The authors suggested that the shared pro-inflammatory milieu of obesity and diabetes may influence CRS severity and recovery, even though a direct causal role of diabetes was not demonstrated. More recent evidence indicates that T2DM and higher fasting blood glucose levels are not only more prevalent among patients with recurrent CRS but also serve as independent predictors of postoperative recurrence, supporting the notion that metabolic status may shape postoperative disease trajectories [49].

Beyond chronic disease, historical clinical observations highlight a propensity toward more severe acute infections in diabetic hosts. Already in 1987, acute bacterial sinusitis in adults with diabetes was described as clinically harder to control, frequently requiring IV antibiotics and, not infrequently, surgical drainage—an experience attributed to impaired neutrophil function and microangiopathy [50].

At the colonization–infection interface, a study published in 2015 [51] showed that while nasal *Staphylococcus aureus* carriage rates in diabetes approximate the general population, carriage in diabetic patients strongly predicts subsequent S. aureus hospitalization, underscoring heightened vulnerability once colonized.

Fungal disease offers an illustration of this vulnerability. In a contemporary cohort spanning the COVID-19 era, a very high DM prevalence was found among invasive fungal rhinosinusitis (especially acute invasive forms), reinforcing diabetes as a dominant risk factor for angioinvasive disease by Mucorales and related species [52]. These observations argue for aggressive, integrated diagnostic pathways in diabetic patients presenting with rapidly progressive sinonasal disease.

Allergic pathways present a more complex, sometimes counterintuitive picture. In a study based on nationally representative data [53], a bidirectional inverse association between AR and DM was observed, while another study published in 2011 [54] showed no link between T1DM and rhinologic allergic disease in twins. Pharmacologic data hint that immune modulation may be operative: in a large propensity-matched cohort of T2DM [55] a lower incidence of AR among users of DPP-4 inhibitors was reported, with a dose–response effect, consistent with CD26-mediated dampening of Th2 activity.

At the genetic level, a study published in 2023 [56] showed no causal signal from T1DM to nasal polyps in bidirectional Mendelian randomization, suggesting that the DM–CRSwNP link seen epidemiologically may reflect type 2-predominant metabolic–inflammatory mechanisms rather than autoimmunity.

Collectively, epidemiology and clinical series converge on a model in which type 2–skewed metabolic inflammation and host–defence impairment in diabetes are associated with

1. CRSwNP susceptibility;
2. Altered sinonasal microbiology with Gram-negative enrichment;
3. Greater severity of bacterial or fungal infections.

while surgical benefit remains achievable with optimized care.

Allergic phenotypes appear to not be increased (and may even be inversely associated) with antidiabetic immunomodulators potentially protective against AR.

**Figure 2:**
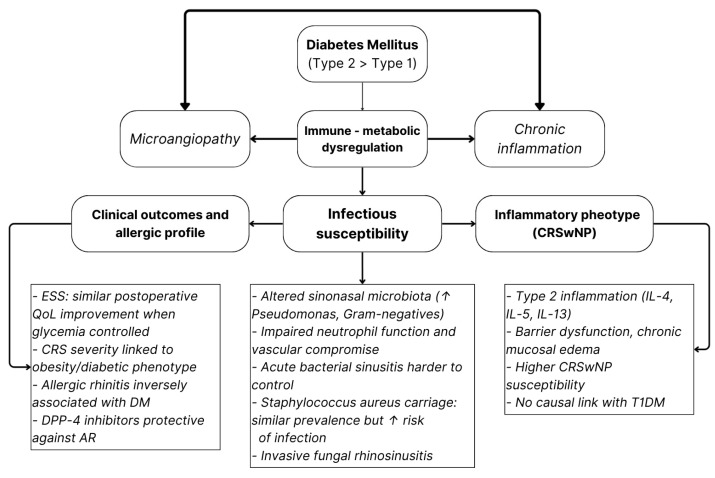
Conceptual model illustrating how DM—particularly type 2—impacts sinonasal health through immune–metabolic dysregulation. Arrows depict the proposed multidirectional interactions and downstream effects linking metabolic imbalance to inflammation, infectious susceptibility, and CRS phenotype [45,46,47,48,49,50,51,52,53,54,55].

A schematic overview of the proposed pathophysiologic links between diabetes and rhinologic disease is shown in Figure 2. The model highlights how metabolic inflammation and vascular compromise may promote CRSwNP susceptibility, microbiota alterations, and infectious vulnerability.

Studies focusing on the association between DM and inflammatory or infectious sinonasal diseases are summarized in Table 1.

A total of 12 studies were identified.

The column “Association direction” indicates whether the study found a significant or suggestive relationship between diabetes and the rhinologic condition (Positive) or no/inverse association (Negative). Specifically, “Positive” denotes a higher prevalence, severity, or worse outcomes of sinonasal disease among diabetic patients, while “Negative” refers to the absence of an association or an inverse relationship (i.e., lower prevalence or milder disease in diabetic patients).

### 3.2. Olfactory Disorders

Olfaction sits at the crossroads of local sinonasal inflammation and systemic metabolic–vascular health. In CRS cohorts, diabetes mellitus independently predicted poorer olfactory performance, reflected by lower TDI and odor identification scores, even after accounting for the dominant effect of nasal polyps [57]. This finding is consistent with evidence of more prolonged olfactory dysfunction in diabetic patients with CRS [45].

Population-based studies underscore the importance of distinguishing crude from adjusted associations. Analyses of KNHANES data showed that the apparent excess of self-reported olfactory dysfunction (OD) in individuals with diabetes mellitus disappears after multivariable adjustment, suggesting confounding by concomitant rhinologic and vascular factors [58]. In a large Swedish population-based cohort of 1387 adults, diabetes was not associated with overall olfactory impairment but was significantly linked to complete anosmia (OR = 2.6), indicating a selective predisposition to severe olfactory loss alongside established factors such as nasal polyps, older age, and male sex [59]. Longitudinal data with follow-up of up to 12 years further demonstrated that DM is associated with a faster decline in odour identification, independent of age and vascular comorbidity, supporting the concept of an accelerated neurosensory trajectory in diabetes [60].

Earlier population studies suggested that OD correlates more strongly with macrovascular disease than with glycaemic control or microvascular complications, raising the possibility that ischemic involvement of olfactory pathways plays a substantive role [61]. Consistent with this interpretation, metabolic analyses from NHANES identified insulin resistance, as measured by HOMA-IR, rather than hyperglycaemia per se, as the factor most closely associated with smell loss in older adults [62].

Clinical observations further indicate that chemosensory dysfunction may reflect disease burden: higher rates of phantosmia and more severe olfactory impairment have been reported among insulin-treated individuals, with greater dysfunction observed in those receiving more intensive therapy [63]. In older adults, T2DM has been identified as an independent risk factor for OD and incorporated as a key predictor in the simplified four-item Concise Aging adults Smell Test (4-CAST) screening tool [64]. Moreover, among elderly outpatients with T2DM, poorer odour identification has been associated with lower Mini-Mental State Examination scores, highlighting the neurocognitive relevance of olfactory testing in this population [65].

In line with the broader impact of diabetes on chemosensory pathways, adults with T1DM have been shown to exhibit markedly reduced olfactory performance, with hyposmia affecting up to 70% of patients [66]. The observed associations with peripheral neuropathy and retinopathy further support the interpretation of OD as part of the wider neurovascular spectrum of diabetic complications.

Taken together, the available evidence supports diabetes mellitus—particularly insulin-resistant, vascularly burdened phenotypes—as a permissive risk environment for olfactory deterioration, shaped by the combined influence of local CRS-related inflammation, systemic macrovascular compromise, and altered metabolic signalling. From a clinical perspective, olfactory assessment may represent a low-cost sentinel marker of broader micro- and macro-organ involvement in diabetic patients (Table 2).

### 3.3. Other Rhinologic Manifestations

Beyond CRS and OD, diabetes interfaces with several additional rhinologic endpoints. Evidence from a large retrospective cohort identified diabetes as a modest but statistically significant risk factor for recurrent spontaneous epistaxis, plausibly mediated by vascular fragility and atherosclerotic changes within the nasal mucosa, in keeping with the broader vascular paradigm discussed above [67]. Mucosal clearance may also be impaired in diabetic patients: assessment of nasal mucociliary clearance using the saccharin transit test demonstrated that diabetes mellitus, particularly when coexisting with hypertension, is independently associated with delayed nasal mucociliary transport, a dysfunction that may enhance susceptibility to infection and promote persistent inflammation [68].

Cough phenotypes in older adults appear sensitive to glycaemic control. In community-dwelling seniors, uncontrolled DM (HbA1c ≥ 8%) was independently associated with frequent, chronic-persistent, and nocturnal cough, even after adjusting for AR, asthma, and smoking, supporting a link between hyperglycaemia, airway inflammation/hyperresponsiveness, and symptom burden [69]. In the COVID-19 setting, anosmia was reported as a relatively frequent symptom overall but without specific associations between DM and rhinologic symptoms [70]; thus, existing data do not establish a DM-specific predisposition to COVID-related smell loss.

Large-scale population-based survey data further showed that DM, without specification of type, was reported as a comorbidity in 10.9% of individuals who contracted COVID-19, within a cohort of 15,166 adults assessed across multiple regions. Among infected participants, anosmia and ageusia were among the most frequently reported symptoms, affecting 68.4% of cases; however, no analyses specifically addressed the relationship between diabetes and olfactory or gustatory dysfunction [71].

Vascular integrity, epithelial transport, and systemic metabolic control likely converge to influence bleeding risk, mucosal clearance, and cough in DM. Evidence remains heterogeneous, but signals are consistent with micro-/macrovascular and metabolic contributions across these domains.

### 3.4. Overall Conclusions and Implications

Taken together, the available literature supports a convergent interpretative framework in which DM, particularly T2DM, affects rhinologic health through three interrelated mechanisms: (1) immune–metabolic dysregulation promoting chronic mucosal inflammation and altered sinonasal microbiology; (2) vascular and neurosensory impairment contributing to progressive olfactory dysfunction and a shift toward more severe phenotypes; and (3) epithelial and vascular mucosal alterations that impair clearance and increase tissue fragility. Within this context, surgical outcomes appear generally comparable to those of non-diabetic patients, provided that metabolic status and systemic risk factors are adequately considered, underscoring the potential relevance of integrated metabolic and rhinologic management.

A global summary of the main findings of all the studies included in this narrative review is provided in Table 3.

## 4. Discussion

A specific limitation of this narrative review is the restriction of the literature search to PubMed. We acknowledge that relevant studies indexed exclusively in other databases may have been missed; however, PubMed was deliberately selected as the primary source for this narrative review because it provides comprehensive coverage of peer-reviewed biomedical and clinical research and ensures standardized indexing through MeSH terms.

### 4.1. Methodological Limitations of the Current Evidence

Despite the growing number of studies exploring the relationship between DM and rhinologic conditions, several methodological limitations substantially weaken the strength and generalizability of the available evidence.

A major limitation is the marked heterogeneity of study designs and populations. Most available evidence derives from cross-sectional or retrospective cohort studies, which inherently preclude causal inference. Only a limited number of investigations adopted prospective or longitudinal designs capable of assessing temporal relationships. Furthermore, study populations range from small tertiary-centre cohorts to large national health surveys, resulting in variable diagnostic accuracy and potential sampling bias.

This lack of robust evidence is particularly evident in the field of OD. A recent scoping review [72] identified only three eligible studies out of more than 3600 screened records, highlighting the extreme paucity of data despite the high prevalence of OD among patients with diabetes. A broader narrative review [73], although summarizing decades of research on DM and OD, similarly reinforces how limited and fragmented the evidence base remains.

### 4.2. Variability in Diabetes Definition and Metabolic Characterization

Another critical limitation concerns the definition of diabetes itself. In several studies, diabetes was self-reported [45,57,58,59,62], relying on participant recall or previous physician diagnosis without biochemical confirmation. Even when laboratory parameters were included, diagnostic thresholds and classification between T1DM and T2DM were often unclear (19 of the 27 studies included in this review), limiting phenotype-specific interpretation. Measures of glycemic control (HbA1c) and disease duration were incorporated in only a minority of studies and were inconsistently associated with outcomes.

### 4.3. Inconsistency in Rhinologic Outcome Assessment

Similarly, the definition and assessment of rhinologic outcomes varied widely across studies. Many population-based investigations [53,58] relied on self-reported symptoms or prior diagnoses, whereas only a limited number employed objective diagnostic criteria, imaging, or validated questionnaires. This issue is particularly relevant for OD, which was frequently assessed subjectively rather than through psychophysical testing such as Sniffin’ Sticks or the Smell Identification Test. As a result, the true prevalence and severity of olfactory impairment in diabetic populations may be either over- or underestimated.

### 4.4. Confounding Factors and Population Heterogeneity

Inadequate control of confounding factors represents an additional source of variability. While some studies adjusted for key metabolic and vascular covariates, others failed to account for important mediators such as obesity, hypertension, smoking, or chronic inflammation, all of which independently influence sinonasal health. The extent of adjustment often determined whether associations between diabetes and rhinologic outcomes remained statistically significant [58].

Geographic and ethnic variability further complicates interpretation. The majority of epidemiological evidence in this narrative review originates from Asian populations, particularly from East Asia, with relatively few studies conducted in Western countries.

Genetic susceptibility, environmental exposure, dietary habits, prevalence of metabolic comorbidities, and healthcare access differ substantially across regions and ethnic groups and may modulate both diabetes phenotype and rhinologic disease expression. Consequently, the magnitude and clinical relevance of observed associations may not be directly generalizable to other populations.

Finally, inter-study inconsistency in diagnostic tools, outcome reporting, and follow-up duration limits meta-analytic synthesis. Standardized instruments such as SNOT-22 or validated olfactory tests were infrequently used, and postoperative or longitudinal outcomes were rarely correlated with metabolic control.

### 4.5. Pathophysiological Framework and Interpretative Considerations

Beyond individual rhinologic phenotypes, the available evidence supports a convergent pathophysiological framework linking diabetes to sinonasal disease through immune–metabolic dysregulation, vascular impairment, and epithelial barrier dysfunction.

Chronic low-grade inflammation and insulin resistance alter innate and adaptive immune responses, promoting persistent mucosal inflammation, impaired host defense, and increased susceptibility to infection.

In parallel, diabetes-related micro- and macrovascular damage may compromise mucosal perfusion and neural integrity, contributing to delayed tissue repair, impaired mucociliary clearance, and accelerated olfactory decline. Epithelial barrier dysfunction further facilitates microbial dysbiosis and perpetuates local inflammation. This triad mirrors the systemic impact of diabetes on organs such as the retina, kidney, and peripheral nervous system, supporting the concept of rhinologic involvement as part of a multisystem disease continuum rather than an isolated manifestation.

### 4.6. Clinical Implications and Future Directions

Although multiple associations between DM and rhinologic manifestations have been reported, causal relationships remain partially unestablished. In this context, emerging human evidence suggests that the nasal microbiota may represent an active biological interface between systemic metabolic alterations and local sinonasal inflammation, with disease-specific and metabolically sensitive microbial signatures being associated with inflammatory severity and mucosal dysfunction [74,75].

In addition, a systematic and longitudinal assessment of the nasal microbiological profile may represent a promising research direction, allowing evaluation of how sinonasal microbial communities evolve over time in relation to glycaemic control, metabolic status, and disease progression.

The integration of metabolic and inflammatory biomarkers, including HbA1c, markers of insulin resistance, systemic inflammatory mediators, and indicators of endothelial dysfunction, may help clarify dose–response relationships and temporal associations.

Advanced imaging techniques, such as high-resolution MRI or functional imaging of olfactory pathways, may further elucidate microvascular and neurosensory involvement.

The emerging evidence also supports the potential role of multidisciplinary management strategies integrating endocrinology and otorhinolaryngology. Olfactory testing represents a low-cost, non-invasive tool that could be incorporated into routine assessment of diabetic patients, potentially serving as an early marker of neurovascular or systemic involvement, analogous to screening approaches used for diabetic retinopathy or neuropathy. Optimized metabolic control should be considered an integral component of rhinologic management, particularly in patients with chronic rhinosinusitis or olfactory dysfunction, alongside standard medical or surgical treatments.

Future research should move beyond cross-sectional and retrospective designs toward well-powered prospective and longitudinal studies capable of capturing temporal relationships between metabolic control and rhinologic outcomes.

Large cohort studies with adequate follow-up would allow evaluation of disease progression, treatment response, and causal pathways.

The systematic adoption of standardized and validated rhinologic assessment tools, such as SNOT-22 and psychophysical olfactory tests, is essential to improve comparability across studies.

Clear differentiation between T1DM and T2DM is crucial, as these conditions differ substantially in pathophysiology, inflammatory burden, and vascular involvement, and may require phenotype-specific preventive and therapeutic strategies.

## 5. Conclusions

While several experimental and mechanistic studies have investigated diabetes-related biological pathways, the body of clinically oriented and methodologically comparable human evidence specifically focused on rhinologic manifestations remains limited. Over nearly five decades of available research, only a limited number of original studies have explored this intersection, underscoring a substantial gap in research relative to the global burden of diabetes.

The few available investigations, although heterogeneous in design and population, converge on the notion that diabetes—particularly T2DM—affects sinonasal health through intertwined immune–metabolic, vascular, and epithelial mechanisms.

These mechanisms appear to

1. Promote CRSwNP susceptibility and alter sinus microbiota toward Gram-negative enrichment;
2. Accelerate olfactory decline via vascular and neuro-sensory compromise. Mirroring the established multidisciplinary pathways used to detect retinopathy in diabetes, olfactory testing might emerge as a similarly valuable tool, one that could be implemented early, perhaps even before symptom onset, to identify individuals at risk of neuro-sensory decline;
3. Contribute to secondary rhinologic issues such as recurrent epistaxis, impaired mucociliary clearance, and increased infection severity.

Future research should address current methodological limitations, particularly the lack of standardized diagnostic tools, small sample sizes, and insufficient differentiation between T1DM and T2DM. Well-powered prospective studies integrating metabolic parameters with objective rhinologic assessments are needed to clarify causal mechanisms. Recognizing rhinologic manifestations as an underexplored component of diabetes may also support earlier detection of systemic vascular and neural involvement and foster multidisciplinary management strategies.

## Figures and Tables

**Table 1:** Association between diabetes mellitus and inflammatory/infectious sinonasal diseases.

Study	Year	N. of Subjects	Results	Type of Diabetes	Association Direction
Nam et al. [45]	2022	34,670 (2608 with DM)	Significant association between DM and CRSwNP, but not with CRS without polyps. CRS patients with DM showed more nasal obstruction and olfactory dysfunction.	Clinically diagnosed DM (not specified)	Positive (for CRSwNP)
Lee et al. [53]	2021	29,246	Bidirectional inverse association between DM and allergic rhinitis: AR patients had lower odds of DM, and DM patients had lower odds of AR, consistent after adjustment.	Mainly T2DM (participants ≥ 30 years)	Negative
Chen et al. [55]	2019	12,408 (6204 DPP-4 inhibitor users; 6204 controls)	DPP-4 inhibitor use in T2DM patients was associated with a lower incidence of allergic rhinitis (aHR = 0.81). High cumulative doses showed stronger protection.	T2DM	Negative (DPP-4 users less AR)
Hajjij et al. [47]	2015	40 (20 DM; 20 controls)	DM did not affect postoperative outcomes of ESS in CRS patients. Both DM and controls had similar improvement in symptoms and QOL.	Mixed (insulin- and non-insulin-dependent)	Negative (no impact of DM on CRS outcomes)
Hart et al. [51]	2015	660	Nasal *S. aureus* carriage prevalence in diabetes similar to general population; however, carriers had a higher risk of subsequent *S. aureus* infection.	Mostly T2DM (97.1%)	Negative (no higher nasal carriage in DM)
Chen et al. [56]	2023	GWAS/UK Biobank data (exact N not stated)	Mendelian randomization showed no causal relationship between T1DM and nasal polyps in either sex.	T1DM	Negative
Soundarya et al. [52]	2025	85	Diabetes was the most common comorbidity, especially linked to invasive fungal rhinosinusitis (AIFRS, CIFRS). Diabetic patients had markedly higher risk and severity of invasive disease.	Not specified	Positive (for invasive FRS)
Jackson et al. [50]	1987	15	Diabetic patients with acute bacterial sinusitis had more severe and persistent infections, often requiring IV antibiotics and drainage; infections not more frequent but clinically more severe.	Adult-onset IDDM and NIDDM (T1DM and T2DM)	Positive (for severity of bacterial sinusitis)
Yuan et al. [49]	2025	1163 CRS patients (134 with T2DM; 276 with post-operative recurrence)	T2DM was significantly more common in patients with postoperative recurrence; T2DM was an independent risk factor for recurrence.	T2DM	Positive
Zhang et al. [46]	2014	376 (19 with DM)	DM associated with higher rates of *P. aeruginosa* and other Gram-negative bacteria in CRS, and poorer QOL improvement after FESS.	Predominantly T2DM (90%)	Positive (worse outcomes and altered microbiology in CRS)
Thomsen et al. [54]	2011	54,530 twins (143 with T1DM)	No significant association between T1DM and hay fever or rhinoconjunctivitis in children or adults.	T1DM	Negative
Steele et al. [48]	2015	241	Diabetes more frequent among obese CRS patients. Both obesity and DM may contribute to chronic sinonasal inflammation; QOL improvement after ESS present but relatively reduced in obese/diabetic individuals.	Type 1 and 2 DM (comorbidity)	Positive (suggested contribution to CRS inflammation)

**Table 2:** Association between diabetes mellitus and olfactory disorders.

Reference	Year	N° of Subjects	Results	Association
Schlosser et al. [57]	2020	388 (224 CRS; 164 controls)	Diabetes (type 1/type 2) independently associated with worse olfactory scores (lower TDI and identification); contributes to olfactory loss in CRS.	Positive
Roh et al. [58]	2021	20,016	Diabetes (both type 1 and type 2 not distinguished) associated with OD in unadjusted analysis, but not after multivariable adjustment.	Negative
Brämerson et al. [59]	2004	1387	Diabetes (type not specified; self-reported presence/absence) not linked to general OD, but significantly associated with anosmia (OR 2.6).	Positive (for anosmia only)
Ekström et al. [60]	2020	1780	Diabetes (type 1 and type 2 combined) predicted faster decline in odour identification over time, independent of other factors.	Positive
Weinstock et al. [61]	1993	111	Diabetic patients (both type 1 and type 2) showed reduced odour identification; impairment related to macrovascular disease, not glycaemic control.	Positive
Min et al. [62]	2018	978	Diabetes type 2/severe insulin resistance doubled the risk of smell dysfunction; no link with fasting glucose or HbA1c.	Positive
Chan et al. [63]	2017	3151	Insulin-treated diabetics had higher odds of phantosmia and severe OD; more intensive therapy correlated with worse smell function. Type 1 and type 2 DM not distinguished.	Positive
Soler et al. [64]	2025	337 (190 development; 147 validation)	Type 2 DM was an independent predictor of OD in older adults (OR ≈ 3.7).	Positive
Sanke et al. [65]	2014	250	High prevalence of OD in elderly Type 2 DM; worse odour identification correlated with cognitive decline; no non-diabetic controls.	Positive (within diabetic cohort)
Falkowski et al. [66]	2017	112 total	T1DM: higher prevalence of hyposmia compared with healthy controls (70% vs. 45.5%)	Positive

**Table 3:** Global findings among the studies included.

Study	Study Type	Population	Type of Diabetes	Investigated Rhinologic Condition
**Nam et al. (2022)** [45]	Cross-sectional, population-based	34,670 patients	Not specified (physician-diagnosed, treated with oral antidiabetic drugs or insulin)	CRSwNP, CRSsNP, OD
**Lee et al. (2021)** [53]	Nationwide cross-sectional, population-based	29,246 adults (≥30 yrs)	Mainly T2DM (participants under 30 excluded)	AR, self-reported physician-diagnosed
**Schlosser et al. (2020)** [57]	Prospective, multi-institutional case-control	388 (224 CRS; 164 controls)	T1DM and T2DM (self-reported, analysed together)	CRSwNP, CRSsNP; OD
**Yuan et al. (2025)** [49]	Retrospective cohort study	1163 adult CRS patients undergoing surgery	T2DM	Postoperative recurrence of CRS
**Roh et al. (2021)** [58]	Population-based cross-sectional	20,016 adults (≥40 yrs)	Not distinguished (self-reported, medication use, or fasting glucose ≥126 mg/dL)	OD; rhinitis and rhinosinusitis as confounders
**Chen et al. (2019)** [55]	Retrospective cohort (propensity score–matched)	12,408 (6204 DPP-4 users; 6204 controls)	T2DM	AR, ICD-9-coded physician diagnosis
**Hajjij et al. (2015)** [47]	Prospective nested case-control within multicenter cohort	40 (20 DM; 20 matched controls) from a total cohort of 473	Both insulin-dependent and non-insulin-dependent diabetes	CRS undergoing endoscopic sinus surgery
**Hart et al. (2015)** [51]	Community-based cross-sectional with prospective follow-up	660	Predominantly T2DM (97.1%)	Nasal *Staphylococcus aureus* colonization (including MRSA)
**Ekström et al. (2020)** [60]	Prospective longitudinal population-based cohort	1780 (dementia-free adults with ≥2 follow-ups over 12 years)	Mainly T2DM (7.6% prevalence)	OD (odour identification decline, Sniffin’ Sticks test)
**Chen et al. (2023)** [56]	Two-sample bidirectional Mendelian randomization	Large GWAS and UK Biobank datasets (European ancestry; sex-stratified)	T1DM	CRSwNP
**Chan et al. (2017)** [63]	Cross-sectional, population-based (NHANES 2013–2014)	3151 adults (≥40 yrs, multiethnic)	Predominantly T2DM (biochemical or physician-diagnosed)	OD (hyposmia, anosmia, phantosmia; 8-item smell test and self-report)
**Soundarya et al. (2025)** [52]	Retrospective observational (tertiary hospital)	85 patients with fungal rhinosinusitis (FRS)	Not specified (likely Type 2; 95% of AIFRS cases diabetic)	Fungal rhinosinusitis (invasive and non-invasive forms: AIFRS, CIFRS, CGFRS, AFRS, fungal ball)
**Weinstock et al. (1993)** [61]	Cross-sectional observational	111 diabetic patients (VA and SUNY centers, USA)	Both T1DM (27%) and T2DM (73%)	OD (odour identification impairment, OCM test)
**Jackson & Rice (1987)** [50]	Retrospective case series	15 diabetic patients (26–71 yrs; 10 IDDM, 5 NIDDM)	Both T1DM (insulin-dependent) and T2DM (non-insulin-dependent)	Acute bacterial sinusitis (mostly maxillary; culture-proven)
**Zhang et al. (2014)** [46]	Retrospective cohort	376 CRS patients (19 with DM; mean age 48 ± 13 yrs)	Both T1DM (10%) and T2DM (90%)	CRS undergoing ESS; bacterial culture and QOL outcomes
**Song et al. (2013)** [69]	Cross-sectional, community-based elderly cohort	796 adults (≥65 yrs)	Predominantly T2DM (HbA1c-based definition; uncontrolled ≥8%)	Cough phenotypes (frequent, persistent, nocturnal); AR as comorbidity/confounder
**Thomsen et al. (2011)** [54]	Nationwide twin registry cross-sectional study	54,530 Danish twins (ages 3–71; 143 with T1DM)	T1DM	Hay fever and rhinoconjunctivitis (questionnaire-based diagnosis)
**Schaalan et al. (2022)** [71]	Cross-sectional population-based survey	15,166 Egyptian adults (18–71 yrs)	Not specified (10.9% diabetic)	Anosmia and ageusia during COVID-19 infection
**Min & Min (2018)** [62]	Cross-sectional, population-based (NHANES 2013–2014)	978 adults (≥50 yrs)	T2DM (self-reported or glucose ≥ 126 mg/dL); insulin resistance (HOMA-IR)	OD (8-item identification test; score ≤ 5 = dysfunction)
**Proença de Oliveira-Maul et al. (2013)** [68]	Cross-sectional observational	252 (79 healthy; 173 with DM and/or HTN)	Both T1DM and T2DM	Decreased nasal mucociliary clearance (saccharin transit test >12 min)
**Sanke et al. (2014)** [65]	Cross-sectional	250 elderly Japanese patients with T2DM (≥65 yrs; median 72)	T2DM	OD (odour identification via Open Essence test)
**Abrich et al. (2014)** [67]	Retrospective cohort	1373 total (461 recurrent epistaxis; 912 controls)	Both T1DM and T2DM	Recurrent spontaneous epistaxis
**Falkowski et al. (2017)** [66]	Cross-sectional observational	112 total (90 T1DM; 22 healthy controls)	T1DM	OD (Sniffin’ Sticks identification test)
**Steele et al. (2015)** [48]	Prospective, multi-institutional observational cohort	241 adults with CRS (66 normal, 76 overweight, 99 obese)	Both T1DM and T2DM (3–11% prevalence depending on BMI)	CRS undergoing ESS; analysis of QOL outcomes by BMI/DM status
**Brämerson et al. (2004)** [59]	Cross-sectional, population-based (Skövde Study, Sweden)	1387 adults (aged ≥20 yrs)	DM (type not specified; self-reported)	OD (hyposmia, anosmia)
**Alasia et al. (2021)** [70]	Prospective, multicentre observational (COVID-19 cohort)	646 hospitalized SARS-CoV-2 positive patients	DM (7.7% diabetic)	Anosmia (12.7%) and rhinorrhoea (3.3%) reported as rhinologic symptoms
**Soler et al. (2025)** [64]	Prospective cross-sectional (development + validation cohorts)	357 total (20 focus group, 190 development, 147 validation)	T2DM non-insulin-dependent	OD (Sniffin’ Sticks and SIT-40 tests)

## Data Availability

No new data were created or analyzed in this study.

## Abbreviations

The following abbreviations are used in this manuscript:

AIFRS	Acute Invasive Fungal Rhinosinusitis
AFRS	Allergic Fungal Rhinosinusitis
AR	Allergic Rhinitis
ABS	Acute Bacterial Sinusitis
BOB	Blood–Olfactory Barrier
BSIT	Brief Smell Identification Test
CD26	Cluster of Differentiation 26
CGFRS	Chronic Granulomatous Fungal Rhinosinusitis
CIFRS	Chronic Invasive Fungal Rhinosinusitis
CRS	Chronic Rhinosinusitis
CRSsNP	Chronic Rhinosinusitis without Nasal Polyps
CRSwNP	Chronic Rhinosinusitis with Nasal Polyps
DALYs	Disability-Adjusted Life Years
DM	Diabetes Mellitus
DPP-4	Dipeptidyl Peptidase-4
ESS	Endoscopic Sinus Surgery
FESS	Functional Endoscopic Sinus Surgery
GWAS	Genome-Wide Association Study
HbA1c	Glycated Hemoglobin
HOMA-IR	Homeostatic Model Assessment for Insulin Resistance
IDDM	Insulin-Dependent Diabetes Mellitus
IL	Interleukin
ILC2	Type 2 Innate Lymphoid Cells
KNHANES	Korea National Health and Nutrition Examination Survey
MRSA	Methicillin-Resistant *Staphylococcus aureus*
NHANES	National Health and Nutrition Examination Survey
NIDDM	Non-Insulin-Dependent Diabetes Mellitus
OD	Olfactory Dysfunction
QOL/QoL	Quality of Life
*S. aureus*	*Staphylococcus aureus*
SIT-40	Smell Identification Test–40
SNOT-22	Sino-Nasal Outcome Test–22
T1D/T1DM	Type 1 Diabetes Mellitus
T2D/T2DM	Type 2 Diabetes Mellitus
Tfh	T Follicular Helper Cells
Th1/Th2/Th17	T Helper Cells
Tregs	Regulatory T Cells
